# Supplementary material for: Modeling glioblastoma heterogeneity as a dynamic network of cell states
Source: Mol Syst Biol. 2021 Sep 16;17(9):e10105. doi: 10.15252/msb.202010105 (PMC8444284; doi:10.15252/msb.202010105)
Supplement: Supplementary file 5 — Source Data for Figure 3 [file MSB-17-e10105-s001.zip › Figure3A_sourcedata/GSEA_3065/hallmarks_state2.GseaPreranked.1623416269049/HALLMARK_UNFOLDED_PROTEIN_RESPONSE.html]

Details for gene set HALLMARK\_UNFOLDED\_PROTEIN\_RESPONSE[GSEA]

|  || Dataset | state2 |
| Phenotype | NoPhenotypeAvailable |
| Upregulated in class | na\_pos |
| GeneSet | HALLMARK\_UNFOLDED\_PROTEIN\_RESPONSE |
| Enrichment Score (ES) | 0.3904536 |
| Normalized Enrichment Score (NES) | 1.3000597 |
| Nominal p-value | 0.08290155 |
| FDR q-value | 0.16854653 |
| FWER p-Value | 0.961 |
Table: GSEA Results Summary

  

Fig 1: Enrichment plot: HALLMARK\_UNFOLDED\_PROTEIN\_RESPONSE      
 Profile of the Running ES Score & Positions of GeneSet Members on the Rank Ordered List

  

| PROBE | GENE SYMBOL | GENE\_TITLE | RANK IN GENE LIST | RANK METRIC SCORE | RUNNING ES | CORE ENRICHMENT || 1 | CKS1B |  |  | 23 | 0.554 | 0.0547 | Yes |
| 2 | LSM4 |  |  | 73 | 0.410 | 0.0918 | Yes |
| 3 | BANF1 |  |  | 198 | 0.283 | 0.1077 | Yes |
| 4 | EIF4EBP1 |  |  | 232 | 0.261 | 0.1311 | Yes |
| 5 | NHP2 |  |  | 274 | 0.242 | 0.1517 | Yes |
| 6 | EXOSC9 |  |  | 298 | 0.233 | 0.1733 | Yes |
| 7 | NPM1 |  |  | 351 | 0.216 | 0.1900 | Yes |
| 8 | NOP56 |  |  | 396 | 0.201 | 0.2061 | Yes |
| 9 | DCP2 |  |  | 474 | 0.184 | 0.2168 | Yes |
| 10 | TUBB2A |  |  | 539 | 0.174 | 0.2280 | Yes |
| 11 | PSAT1 |  |  | 561 | 0.170 | 0.2433 | Yes |
| 12 | DKC1 |  |  | 592 | 0.165 | 0.2571 | Yes |
| 13 | KIF5B |  |  | 604 | 0.163 | 0.2727 | Yes |
| 14 | EIF4A3 |  |  | 635 | 0.158 | 0.2858 | Yes |
| 15 | NFYB |  |  | 740 | 0.145 | 0.2897 | Yes |
| 16 | EIF4A1 |  |  | 765 | 0.142 | 0.3018 | Yes |
| 17 | RPS14 |  |  | 834 | 0.134 | 0.3084 | Yes |
| 18 | CEBPG |  |  | 860 | 0.131 | 0.3193 | Yes |
| 19 | IMP3 |  |  | 991 | 0.121 | 0.3178 | Yes |
| 20 | KHSRP |  |  | 993 | 0.120 | 0.3301 | Yes |
| 21 | HSPA9 |  |  | 996 | 0.120 | 0.3423 | Yes |
| 22 | MTHFD2 |  |  | 1014 | 0.118 | 0.3527 | Yes |
| 23 | EIF4E |  |  | 1038 | 0.116 | 0.3622 | Yes |
| 24 | EIF2S1 |  |  | 1051 | 0.115 | 0.3728 | Yes |
| 25 | LSM1 |  |  | 1088 | 0.112 | 0.3806 | Yes |
| 26 | NOLC1 |  |  | 1104 | 0.111 | 0.3905 | Yes |
| 27 | EXOSC5 |  |  | 1376 | 0.093 | 0.3711 | No |
| 28 | RRP9 |  |  | 1583 | 0.082 | 0.3575 | No |
| 29 | NOP14 |  |  | 1648 | 0.079 | 0.3589 | No |
| 30 | SERP1 |  |  | 1717 | 0.076 | 0.3595 | No |
| 31 | POP4 |  |  | 1736 | 0.076 | 0.3653 | No |
| 32 | PAIP1 |  |  | 2060 | 0.062 | 0.3372 | No |
| 33 | EXOSC2 |  |  | 2108 | 0.061 | 0.3385 | No |
| 34 | CXXC1 |  |  | 2181 | 0.058 | 0.3367 | No |
| 35 | EXOSC4 |  |  | 2221 | 0.056 | 0.3384 | No |
| 36 | SDAD1 |  |  | 2531 | 0.047 | 0.3101 | No |
| 37 | PARN |  |  | 2581 | 0.045 | 0.3095 | No |
| 38 | GEMIN4 |  |  | 2615 | 0.044 | 0.3106 | No |
| 39 | CNOT6 |  |  | 2641 | 0.043 | 0.3123 | No |
| 40 | XPOT |  |  | 3155 | 0.031 | 0.2606 | No |
| 41 | EIF4G1 |  |  | 3559 | 0.023 | 0.2198 | No |
| 42 | DCTN1 |  |  | 3602 | 0.022 | 0.2176 | No |
| 43 | TATDN2 |  |  | 3783 | 0.019 | 0.2003 | No |
| 44 | EXOSC10 |  |  | 3825 | 0.018 | 0.1978 | No |
| 45 | CNOT2 |  |  | 4026 | 0.015 | 0.1779 | No |
| 46 | PREB |  |  | 4188 | 0.012 | 0.1620 | No |
| 47 | EXOSC1 |  |  | 4325 | 0.010 | 0.1485 | No |
| 48 | ALDH18A1 |  |  | 4450 | 0.008 | 0.1360 | No |
| 49 | VEGFA |  |  | 4721 | 0.004 | 0.1075 | No |
| 50 | DCP1A |  |  | 5175 | -0.003 | 0.0593 | No |
| 51 | ERO1A |  |  | 5380 | -0.006 | 0.0382 | No |
| 52 | EXOC2 |  |  | 5533 | -0.009 | 0.0228 | No |
| 53 | ASNS |  |  | 5539 | -0.009 | 0.0232 | No |
| 54 | CEBPB |  |  | 5541 | -0.009 | 0.0240 | No |
| 55 | SLC30A5 |  |  | 5947 | -0.016 | -0.0177 | No |
| 56 | ATF4 |  |  | 5987 | -0.016 | -0.0202 | No |
| 57 | GOSR2 |  |  | 6198 | -0.020 | -0.0406 | No |
| 58 | FUS |  |  | 6216 | -0.020 | -0.0404 | No |
| 59 | ZBTB17 |  |  | 6511 | -0.025 | -0.0692 | No |
| 60 | DDX10 |  |  | 6539 | -0.026 | -0.0694 | No |
| 61 | EEF2 |  |  | 6630 | -0.028 | -0.0762 | No |
| 62 | MTREX |  |  | 6659 | -0.028 | -0.0762 | No |
| 63 | ATF3 |  |  | 6737 | -0.030 | -0.0814 | No |
| 64 | XBP1 |  |  | 6931 | -0.035 | -0.0984 | No |
| 65 | ARFGAP1 |  |  | 7123 | -0.040 | -0.1148 | No |
| 66 | YWHAZ |  |  | 7252 | -0.044 | -0.1240 | No |
| 67 | WFS1 |  |  | 7253 | -0.044 | -0.1195 | No |
| 68 | SLC1A4 |  |  | 7280 | -0.044 | -0.1177 | No |
| 69 | KDELR3 |  |  | 7339 | -0.046 | -0.1192 | No |
| 70 | ATP6V0D1 |  |  | 7376 | -0.047 | -0.1182 | No |
| 71 | SRPRA |  |  | 7388 | -0.047 | -0.1145 | No |
| 72 | TTC37 |  |  | 7469 | -0.050 | -0.1180 | No |
| 73 | EDEM1 |  |  | 7566 | -0.052 | -0.1229 | No |
| 74 | FKBP14 |  |  | 7766 | -0.059 | -0.1381 | No |
| 75 | DNAJB9 |  |  | 7882 | -0.062 | -0.1440 | No |
| 76 | YIF1A |  |  | 7991 | -0.067 | -0.1486 | No |
| 77 | SLC7A5 |  |  | 8067 | -0.070 | -0.1495 | No |
| 78 | BAG3 |  |  | 8104 | -0.071 | -0.1460 | No |
| 79 | SHC1 |  |  | 8385 | -0.088 | -0.1668 | No |
| 80 | SPCS3 |  |  | 8396 | -0.089 | -0.1588 | No |
| 81 | CNOT4 |  |  | 8428 | -0.091 | -0.1527 | No |
| 82 | WIPI1 |  |  | 8505 | -0.096 | -0.1509 | No |
| 83 | SEC11A |  |  | 8575 | -0.101 | -0.1479 | No |
| 84 | DDIT4 |  |  | 8601 | -0.103 | -0.1399 | No |
| 85 | HYOU1 |  |  | 8614 | -0.104 | -0.1304 | No |
| 86 | ATF6 |  |  | 8671 | -0.109 | -0.1252 | No |
| 87 | SPCS1 |  |  | 8717 | -0.113 | -0.1184 | No |
| 88 | HERPUD1 |  |  | 8723 | -0.114 | -0.1072 | No |
| 89 | SSR1 |  |  | 8765 | -0.118 | -0.0994 | No |
| 90 | SEC31A |  |  | 8793 | -0.122 | -0.0897 | No |
| 91 | SRPRB |  |  | 8832 | -0.127 | -0.0807 | No |
| 92 | EIF4A2 |  |  | 9067 | -0.160 | -0.0892 | No |
| 93 | DNAJC3 |  |  | 9150 | -0.177 | -0.0797 | No |
| 94 | PDIA6 |  |  | 9165 | -0.180 | -0.0626 | No |
| 95 | CALR |  |  | 9310 | -0.237 | -0.0536 | No |
| 96 | HSP90B1 |  |  | 9334 | -0.257 | -0.0296 | No |
| 97 | HSPA5 |  |  | 9406 | -0.395 | 0.0036 | No |
Table: GSEA details [plain text format]

  

Fig 2: HALLMARK\_UNFOLDED\_PROTEIN\_RESPONSE: Random ES distribution      
 Gene set null distribution of ES for **HALLMARK\_UNFOLDED\_PROTEIN\_RESPONSE**
